# Effects of combining Shugan Jieyu capsule treatment with group psychological counseling on alexithymia in hemodialysis patients

**DOI:** 10.3389/fphar.2026.1688593

**Published:** 2026-03-05

**Authors:** Xiaojie Gao, Xiaoling Li, Susu Gong, Cuiying Yang, Yujie Hao

**Affiliations:** 1 Hemodialysis Unit, Qian’an People’s Hospital, Qian’an, Hebei, China; 2 Department of Critical Care Medicine, Qian’an People’s Hospital, Qian’an, Hebei, China; 3 Department of Hematology, Xiangya Hospital, Central South University, Changsha, Hunan, China; 4 Emergency Command Center, Qian’an, Hebei, China; 5 Department of Nephrology, Qian’an Hospital of Traditional Chinese Medicine, Qian’an, Hebei, China

**Keywords:** alexithymia, anxiety, depression, group psychological counseling, hemodialysis, Shugan Jieyu capsule

## Abstract

**Aim:**

To explore the effects of combining Shugan Jieyu capsule treatment with group psychological counseling on alexithymia in patients undergoing hemodialysis.

**Methods:**

A total of 120 patients with nephropathy who underwent hemodialysis at our hospital’s dialysis center from January 2023 to December 2024 were enrolled in this study and randomly divided into the control and study groups. The control group received estazolam treatment with routine nursing, whereas the study group received the Shugan Jieyu capsule along with group psychological counseling in addition to estazolam treatment and routine nursing. Various parameters were then measured and compared between the two groups, including alexithymia, depression, anxiety, sleep quality, personal and social performances, self-care ability, quality of life, serum levels of orphanin FQ and interleukin-2, incidence of adverse reactions, and nursing satisfaction.

**Results:**

At both 4 and 8 weeks post-intervention, all measured parameters improved significantly from the baseline in both groups (*p* < 0.05). Importantly, the study group demonstrated better outcomes than the control group, including significantly lower scores for alexithymia, anxiety, depression, and sleep quality as well as higher scores for personal and social performances, self-care ability, and quality of life. Additionally, the serum levels of orphanin FQ and interleukin-2 were significantly lower in the study group (*p* < 0.05).

**Conclusion:**

Shugan Jieyu capsule treatment combined with group psychological counseling was shown to effectively alleviate alexithymia, anxiety, and depression; improve sleep quality; reduce serum levels of orphanin FQ and interleukin-2; and enhance personal and social performances, self-care ability, quality of life, and nursing satisfaction in hemodialysis patients.

## Introduction

Hemodialysis is the main treatment mode for kidney disease, especially end-stage renal disease (Vachharajani et al., 2021). The protracted and demanding nature of this treatment often leads to significant physical discomfort and compromised immunity (Loutradis et al., 2021). Furthermore, repeated vascular access procedures and the substantial financial burden contribute to the high prevalence of psychological distress in the patients, including anxiety and depression, which can adversely affect treatment adherence and outcomes (Wang et al., 2024). Consequently, psychological wellbeing and quality of life are now regarded as critical indicators alongside survival rates in the comprehensive evaluation of patients with end-stage renal disease (Al-Ghabeesh et al., 2022).

Among the array of psychological challenges, alexithymia is a construct first described by Sifneos in the 1970s that is particularly salient yet underaddressed (Hogeveen and Grafman, 2021). Alexithymia is often called “affective dyslexia” and is characterized by difficulties in identifying and describing feelings, an externally oriented thinking style, and limited imaginative capacity (Preece et al., 2023). In the context of chronic illness, alexithymia may arise as a maladaptive coping mechanism in response to persistent stress and somatic symptoms, which is a highly relevant scenario to hemodialysis patients. Individuals with alexithymia tend to confuse emotional distress with physical sensations, leading to somatization and exaggerated somatic complaints that complicate clinical assessment and management (Iskric et al., 2020). Critically, studies have reported high prevalence rates of alexithymia among hemodialysis patients ranging from 13% to 80%, which is significantly higher than in the general population (Pojatić et al., 2022). This condition is strongly associated with elevated levels of anxiety and depression as well as poorer quality of life overall (Holmes et al., 2022). Therefore, developing effective interventions to mitigate alexithymia is imperative for improving the mental health and treatment prognosis of this vulnerable population.

Existing interventions for alexithymia primarily focus on psychotherapeutic approaches. Techniques such as mindfulness-based therapy, cognitive-behavioral therapy adapted for emotional awareness, and psychodynamic therapy have shown promising outcomes in improving emotional processing skills (Li et al., 2025; Tsubaki and Shimizu, 2024). However, their application in hemodialysis settings is limited by various factors like time constraints, post-dialysis patient fatigue, and shortage of specialized mental health professionals. Pharmacological interventions, mainly antidepressants, are commonly used in the treatment of comorbid anxiety and depression but do not target the core deficits of alexithymia directly. This gap highlights the need for accessible and multimodal interventions that can be integrated into routine dialysis care.

In this context, the combination of a traditional Chinese medicine (TCM) formulation with structured group intervention offers a novel and theoretically synergistic approach. Shugan Jieyu capsule is a TCM formulation comprising standardized extracts from *Hypericum perforatum* and *Acanthopanax senticosus* that has demonstrated anxiolytic and antidepressant properties (Zhang and Bai, 2022). Its proposed mechanisms include modulation of monoamine neurotransmitters like serotonin and dopamine, which are implicated in both mood regulation and the neurobiology of alexithymia (Zhang et al., 2024). Although this treatment was shown to be effective in stroke patients (Yao et al., 2022), the specific impacts on the characteristic emotional processing deficits of alexithymia remain unexplored in hemodialysis patients.

Concurrently, group psychological counseling is an ideal platform for addressing the interpersonal and experiential aspects of alexithymia; it fosters a supportive environment in which individuals can safely practice identifying, expressing, and reflecting on emotions through interaction with peers experiencing similar challenges (Zhang et al., 2022). This format directly counteracts the social isolation and emotional constriction observed in alexithymia by fostering a sense of belonging and providing modeled emotional communication. Although group counseling strategies have been reported in other treatment populations (Yin et al., 2023), there have been limited applications to hemodialysis patients.

While standalone TCM or psychological interventions have shown beneficial outcomes for depression and quality of life in patients with chronic kidney disease (Xia et al., 2022), there are scarce research reports on their combined use. Importantly, there are no studies to date that have specifically designed and evaluated a combined intervention targeting the core symptoms of alexithymia in hemodialysis patients. The hypothesized synergy lies in the potential of Shugan Jieyu capsule to ameliorate the neurobiological substrates of emotional blunting, thereby potentially increasing patient receptivity and capacity to engage in the emotional skills training provided via group counseling.

To objectively evaluate the potential biological mechanisms underlying the interventional effects of the combined therapy, we selected two specific serum biomarkers: orphanin FQ (OFQ) and interleukin-2 (IL-2). The rationale for this selection is twofold and aligns with the dominant neurobiological hypotheses of alexithymia. First, the “opioid peptide hypothesis” posits that dysregulation of endogenous opioid systems, particularly OFQ (also known as nociceptin), is involved in stress response and emotional blunting that are core features of alexithymia (Dib et al., 2021). Elevated OFQ levels are associated with reduced emotional responsiveness and may mediate the link between chronic stress and affective deficits (D'Oliveira da Silva et al., 2024). Second, the “inflammatory hypothesis” suggests that chronic low-grade inflammation, reflected by cytokines like IL-2, contributes to neuropsychiatric symptoms by affecting neurotransmitter metabolism and the neural circuitry (Williams et al., 2022). IL-2 is a key immunoregulatory cytokine that has been implicated in the pathophysiology of depression and somatic symptom burden, which are processes closely related to alexithymia (Guilbaud et al., 2021). Therefore, measuring changes in the systemic levels of OFQ and IL-2 provides a window into the neuroimmune pathways that may be modulated by our combined intervention.

To address this significant gap, the present work aims to investigate the efficacy of a combined intervention encompassing Shugan Jieyu capsule treatment and structured group psychological counseling on alexithymia and its associated biopsychosocial outcomes in hemodialysis patients.

## Materials and methods

### Medicinal materials

The Shugan Jieyu capsule used in this study is a commercially available standardized botanical drug product approved by the National Medical Products Administration of China (approval number: Z20080580) and manufactured by Chengdu Kanghong Pharmaceutical Group Co., Ltd. Each capsule contains 0.36 g of a standardized extract derived from *H. perforatum* (aerial parts) and *A. senticosus* (Rupr. and Maxim.) Harms (roots and rhizomes). These botanical components used in the Shufeng Jieyu capsule are all sourced from artificial cultivation and are not endangered species. Their procurement and use also follow ethical guidelines. The manufacturing process and specific extraction parameters are proprietary to the manufacturer. The product quality, including the content ranges of the key marker constituents (e.g., total hypericins from *H. perforatum* and total eleutherosides from *A. senticosus*), is controlled to meet the standards of the Chinese pharmacopoeia (2020 edition).

### Study design

**FIGURE 1:**
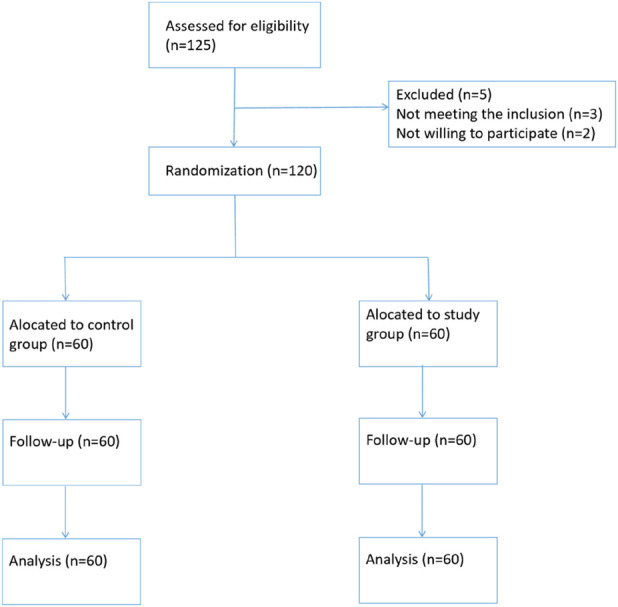
CONSORT flow diagram.

A total of 120 nephropathy patients who underwent hemodialysis at the dialysis center of our hospital from January 2023 to December 2024 were selected as the study participants, and the corresponding flow diagram is shown in Figure 1.

The inclusion criteria for the study are as follows: the patients met the diagnostic criteria for end-stage renal disease, and the glomerular filtration rate was less than 15 mL/min/1.73 m^2^; the duration of diagnosis of the disease course was 6–18 months, and regular dialysis was being performed for more than 6 months; the patients underwent dialysis 2–3 times a week.

The exclusion criteria for the study were as follows: the patients had acute cardiac and cerebrovascular complications; the patients were discharged (transferred) or died after completing the group counseling; the patients did not complete the group counseling or requested to quit; those who were allergic to any of the constituents in the Shugan Jieyu capsule.

Informed consent was obtained from the patients and their families, and this study was approved by the ethics committee of the hospital; the clinical registration number for this study was ChiCTR2300068249.

### Sample size calculation

A convenience sampling technique was employed to recruit potential participants from all eligible patients receiving hemodialysis at the center. The sample size was determined based on a prestudy power analysis (Romeo et al., 2025). Using the score from the Toronto Alexithymia Scale-20 (TAS-20) as the primary endpoint along with an anticipated effect size of 0.65 (based on a pilot study), a two-sided α of 0.05, and a power (1 − β) of 80%, we determined that a minimum of 50 patients per group was required. By accounting for a potential dropout rate of 20%, we aimed to recruit 60 patients per group for a total of 120 patients.

### Randomization and allocation concealment

The patients were randomly divided into the control and study groups, with 60 persons in each group. Randomization was performed using a computer-generated random number sequence. The allocation information was concealed in sequentially numbered, opaque, and sealed envelopes that were opened by a research assistant who was not involved in patient recruitment or outcome assessment after the patient had provided informed consent.

### Blinding of outcome assessment

Given the nature of the interventions (oral medication and psychological counseling), it was not feasible to blind the patients or the nursing staff providing the interventions. However, to minimize assessment bias, all outcome assessments (including all scale evaluations and laboratory tests) were performed by a dedicated research nurse who was blinded to the group allocations of the patients. This nurse was not involved in the delivery of any intervention.

### Treatment methods

All enrolled patients received basic treatment (including blood pressure and blood sugar level controls as well as correction of calcium and phosphorus metabolism and anemia symptoms) and routine hemodialysis (4 h each session, 3 times a week).

The control group was given estazolam (produced by Guangdong Taicheng Pharmaceutical Co., Ltd.) 1 mg once a day orally before bedtime.

Based on the estazolam treatment, the study group was given oral treatment of Shugan Jieyu capsule at a dosage of two capsules twice a day.

Both groups were treated for 4 weeks as a course.

### Nursing methods

The control group received routine nursing. Before the patient’s initial dialysis, the nurse introduced the ward environment and precautions. Before undergoing dialysis, the nurse introduced the treatment effects to the patient and their family in detail as well as guided the patient to cooperate with the treatment procedures, protection of the arteriovenous puncture points, and response and alleviation of adverse reactions during treatment. At the same time, the reactions and adverse emotions of the patient were observed and communicated.

The study group was provided group psychological counseling based on routine nursing as follows:

1. Group psychological counseling teams were set up for intervention and data collection that included one leader (who had received psychiatric nursing training) and two team members (from the health education department). Among them, the team leader had the qualification of being a national second-level psychological counselor, had more experience in group psychological counseling, was familiar with the processes of group psychological counseling, and had mastered the key points of dialysis nursing work. Before commencing the sessions, the team members were systematically trained for 2 d so that they could master the requirements of group therapy. When issuing questionnaires, a unified introduction was used to accurately and objectively explain the purpose of the study to the patients, effectively assist patients with filling in the relevant questionnaires, and assist the group leader with observing patients and recording the group counseling sessions.
2. Patients in the study group were divided into 10 teams of six patients each. The interventions were conducted once a week for 45–60 min each time for a total of 8 weeks. All activities were carried out in a relatively closed demonstration classroom. A total of eight activities were designed, where each activity had a specific theme as follows: (a) introducing patients to each other, obtaining the cooperation of patients, and explaining the intervention procedures (expected goals, subsequent steps, and time and place of counseling); (b) providing a good atmosphere for patients to learn from each other, enhancing patient belief in the treatment, and clarifying the important role of emotion on disease outcome and quality of life to the patients; (c) disseminating knowledge on how to manage discomfort during hemodialysis treatment through cases; (d) teaching communication skills and instructing patients on accurately expressing emotions to others through repeated practice; (e) identifying cognitive problems in patients along with reducing anxiety and depression in dialysis patients; (f) interpersonal training with focus on listening to encourage patients to share their expressions and provide emotional support; (g) organizing well-adapted patients to speak and share their personal psychological experiences, changes in the occurrence and development of the disease, and methods to better cope with problems through expressing emotions and solving problems effectively; (h) the group psychological counseling team members summarize reports and share the changes and experiences over the 8 weeks with the patients. The group psychological counseling sessions mainly involved explanations by the group counselor, discussions between the group members, listening to each other, playing the “family” dialogue, and interactive learning approaches like the personal appearances of the patients and others to help patient participate during the counseling process; these efforts were aimed at achieving the purpose of adapting to the *status quo* as well as improving treatment compliance and quality of life.

### Observation indicators

1. Alexithymia was assessed using the Chinese version of the TAS-20 (Tuliao et al., 2020). This 20-item scale comprises three dimensions: difficulty with identifying feelings, difficulty with describing feelings, and externally oriented thinking. The total score ranges from 20 to 100. According to the established cutoff points for the Chinese version, a total score <50 indicates the absence of alexithymia, while a score ≥61 indicates the presence of alexithymia, and scores of 51–60 suggest possible alexithymia. The Chinese version has demonstrated good reliability, with a reported Cronbach’s α coefficient of 0.83 in the validation study. In the present study, the Cronbach’s α value was 0.85.
2. Anxiety and depression symptoms were measured using the validated Chinese versions of the Hamilton Anxiety Scale (HAMA) and Hamilton Depression Scale (HAMD) (Meng et al., 2022). The total scores for these measures are interpreted as follows: 8–14 indicates mild symptoms, 15–20 indicates moderate symptoms, and ≥21 indicates severe symptoms. The validation studies for the Chinese versions of these tools showed good internal consistency, with Cronbach’s α coefficients of 0.86 for HAMA and 0.88 for HAMD. In our study, the Cronbach’s α values were 0.87 for HAMA and 0.89 for HAMD.
3. Sleep quality was evaluated using the Chinese version of the Pittsburgh Sleep Quality Index (PSQI) (Zitser et al., 2022). The global PSQI scores range from 0 to 21, with higher scores indicating poorer sleep quality. The Chinese version has shown good psychometric properties, with a reported Cronbach’s α of 0.82 in the validation study; this α value in the present study was 0.84.
4. Personal and social performances were assessed using the Chinese version of the Personal and Social Performance (PSP) scale (Rabinowitz et al., 2021). The scores for this tool range from 0 to 100, with higher scores reflecting better functioning. This instrument has previously demonstrated adequate reliability in the Chinese population, with a reported Cronbach’s α >0.80.
5. Self-care ability was measured using the Chinese version of the Exercise of Self-Care Agency (ESCA) scale (Cunha et al., 2024). This 172-point instrument encompasses four subscales, namely, self-concept (0–36), self-care skills (0–48), health knowledge (0–56), and self-care responsibility (0–32), where higher total scores indicate greater self-care ability. The Chinese version was shown to have good internal consistency, with the validation study reporting a Cronbach’s α of 0.89.
6. Quality of life was evaluated using the Chinese version of the World Health Organization Quality of Life-BREF (WHOQOL-BREF) (Mamipour et al., 2023); this instrument yields scores in four domains, namely, physical health, psychological health, social relationships, and environment. Each domain’s score is interpreted on a scale of 0–100, with higher scores denoting better quality of life. This tool has been widely used and validated in China and has shown good internal consistency across the domains, with the Cronbach’s α values ranging from 0.76 to 0.88.
7. To assess the serum levels of the biomarkers of interest, 5 mL of fasting venous blood was collected from each participant. The serum OFQ concentration was measured using a commercial radioimmunoassay kit, while the serum IL-2 level was determined by an immunoluminescence assay. All assays were performed in duplicate according to manufacturer protocols. The intra- and inter-assay coefficients of variation for both assays were less than 10%.
8. The incidence of adverse events (e.g., dizziness, drowsiness, and dry mouth/thirst) was actively monitored and recorded throughout the study period in both groups.
9. At the end of intervention, we assessed patient satisfaction with nursing care using a self-administered questionnaire developed by our hospital. The questionnaire included items related to the professionalism of staff, quality of care, and perceived effectiveness of care and yields a total score out of 100. Satisfaction was categorized into different levels as follows: very satisfied (≥90), satisfied (60–89), and dissatisfied (<60). The overall satisfaction rate was calculated as [(number of very satisfied respondents + number of satisfied respondents)/total number of respondents] × 100%. The internal consistency of this questionnaire in the present study was acceptable, with a Cronbach’s α value of 0.79.

### Statistical analysis

Statistical analyses were performed using SPSS 25.0 software. The continuous data were presented as mean ± standard deviation (x ± s), while the categorical data were expressed as numbers and percentages (%). To compare the baseline characteristics between the two groups, independent sample t-tests were used for the continuous variables and chi-squared (χ^2^) tests were used for the categorical variables. For the outcome measures assessed at baseline, 4 weeks, and 8 weeks, a two-way repeated-measures analysis of variance (ANOVA) was employed to examine the effects of time, group, and time × group interaction. If a significant interaction effect was found, simple effects analyses were conducted to compare the groups at each time point and to compare the time points within each group. For post-hoc pairwise comparisons, we used the Tukey test to adjust for multiple comparisons. The incidence of adverse reactions and nursing satisfaction rates were compared between groups using the χ^2^ test as needed. A two-tailed *p*-value of <0.05 was considered statistically significant.

## Results

### Baseline data of patients in the two groups

Table 1 shows a comparison of the general information between the two groups, which did not yield any results of statistical significance (*p* > 0.05).

**TABLE 1:** General information on the patients in the two groups.

Characteristic	Control group (n = 60)	Study group (n = 60)	χ^2^/t	*p*-value
Gender, n (%)				
Male	30 (50.00)	27 (45.00)	0.30	0.58
Female	30 (50.00)	33 (55.00)		
Age (years), n (%)	45.35 ± 5.36	45.41 ± 5.42	0.06	0.95
Education level, n (%)				
Primary school or below	12 (20.00)	15 (25.00)	0.85	0.65
Middle/high school	36 (60.00)	32 (53.33)		
College or above	12 (20.00)	13 (21.67)		
Marital status, n (%)				
Married	48 (80.00)	51 (85.00)	1.07	0.59
Unmarried/single	8 (13.33)	5 (8.33)		
Others (divorced/widowed)	4 (6.67)	4 (6.67)		
Employment status, n (%)				
Employed	15 (25.00)	17 (28.33)	0.27	0.60
Unemployed/retired	45 (75.00)	43 (71.67)		
Primary category of renal disease, n (%)				
Diabetic nephropathy	25 (41.67)	22 (36.67)	1.58	0.66
Hypertensive nephropathy	18 (30.00)	20 (33.33)		
Chronic glomerulonephritis	12 (20.00)	15 (25.00)		
Others	5 (8.33)	3 (5.00)		
Dialysis duration (months)	10.53 ± 2.35	10.35 ± 2.30	0.42	0.67

### Patient alexithymia between the two groups

**FIGURE 2:**
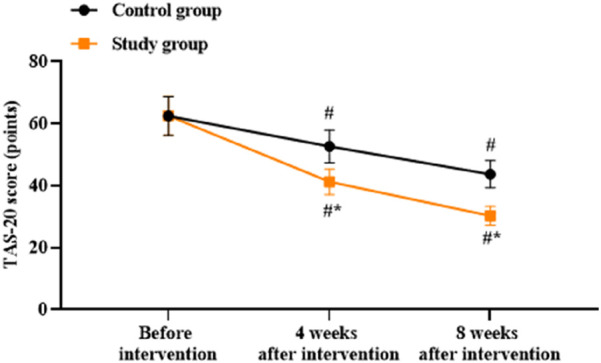
Patient alexithymia scores in both groups. ^#^ *p* < 0.05 vs. before intervention; ^*^ *p* < 0.05 vs. control group.

There were no significant differences in the TAS-20 scores between the two groups before intervention (*p* > 0.05). Compared to before intervention, the TAS-20 scores of both groups were lower at 4 and 8 weeks after intervention (*p* < 0.05). Compared to the control group, the study group had lower TAS-20 scores at 4 and 8 weeks after intervention (*p* < 0.05, Figure 2).

### Patient anxiety and depression between the two groups

**FIGURE 3:**
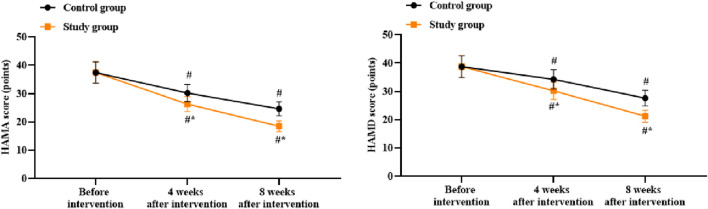
Patient anxiety (HAMA) and depression (HAMD) scores in both groups. ^#^ *p* < 0.05 vs. before intervention; ^*^ *p* < 0.05 vs. control group.

There were no significant differences in the HAMA and HAMD scores between the two groups before intervention (*p* > 0.05). Compared to before intervention, the HAMA and HAMD scores of both groups were lower at 4 and 8 weeks after intervention (*p* < 0.05). Compared to the control group, the study group had lower HAMA and HAMD scores at 4 and 8 weeks after intervention (*p* < 0.05, Figure 3).

### Patient sleep quality between the two groups

**FIGURE 4:**
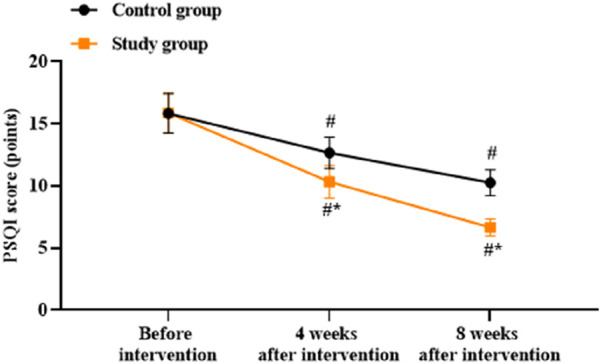
Patient sleep quality based on the Pennsylvania Sleep Quality Index (PSQI) in both groups. ^#^ *p* < 0.05 vs. before intervention; ^*^ *p* < 0.05 vs. control group.

There was no significant difference in the PSQI scores between the two groups before intervention (*p* > 0.05). Compared to before intervention, the PSQI scores of both groups were lower at 4 and 8 weeks after intervention (*p* < 0.05). Compared to the control group, the study group had lower PSQI scores at 4 and 8 weeks after intervention (*p* < 0.05, Figure 4).

### Patient personal and social performances between the two groups

**FIGURE 5:**
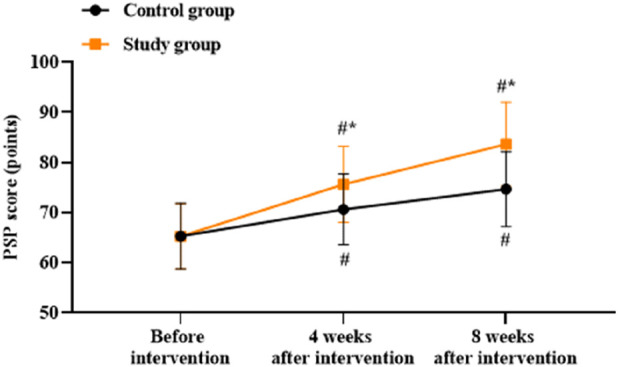
Patient Personal and Social Performance (PSP) scores in both groups. ^#^ *p* < 0.05 vs. before intervention; ^*^ *p* < 0.05 vs. control group.

There was no significant difference in the PSP scores between the two groups before intervention (*p* > 0.05). Compared to before intervention, the PSP scores of both groups were higher at 4 and 8 weeks after intervention (*p* < 0.05). Compared to the control group, the study group had higher PSP scores at 4 and 8 weeks after intervention (*p* < 0.05, Figure 5).

### Patient self-care abilities between the two groups

**FIGURE 6:**
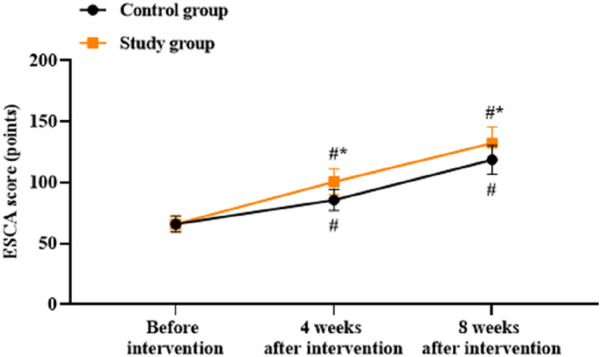
Patient self-care abilities based on the Exercise of Self-Care Agency (ESCA) scores in both groups. ^#^ *p* < 0.05 vs. before intervention; ^*^ *p* < 0.05 vs. control group.

There was no significant difference in the ESCA scores between the two groups before intervention (*p* > 0.05). Compared to before intervention, the ESCA scores of both groups were higher at 4 and 8 weeks after intervention (*p* < 0.05). Compared to the control group, the study group had higher ESCA scores at 4 and 8 weeks after intervention (*p* < 0.05, Figure 6).

### Patient quality of life between the two groups

**FIGURE 7:**
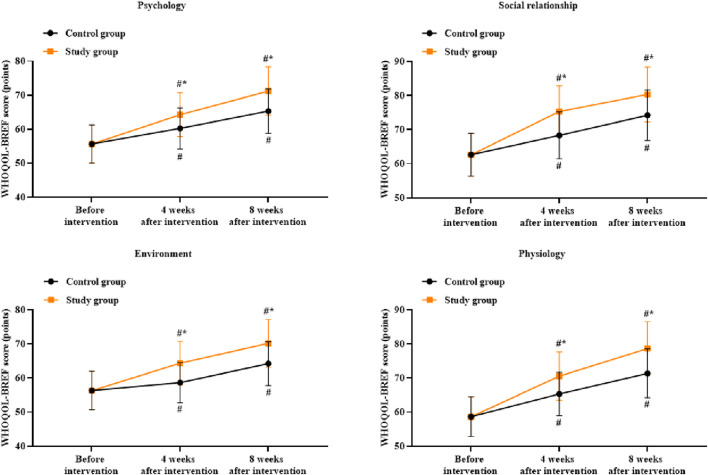
Patient quality of life based on four dimensions in both groups. ^#^ *p* < 0.05 vs. before intervention; ^*^ *p* < 0.05 vs. control group.

There were no significant differences in the WHOQOL-BREF scores between the two groups before intervention (*p* > 0.05). Compared to before intervention, the WHOQOL-BREF scores of both groups were higher for all domains at 4 and 8 weeks after intervention (*p* < 0.05). Compared to the control group, the study group had higher WHOQOL-BREF scores at 4 and 8 weeks after intervention (*p* < 0.05, Figure 7).

### Serum levels of OFQ and IL-2 between the two groups

**FIGURE 8:**
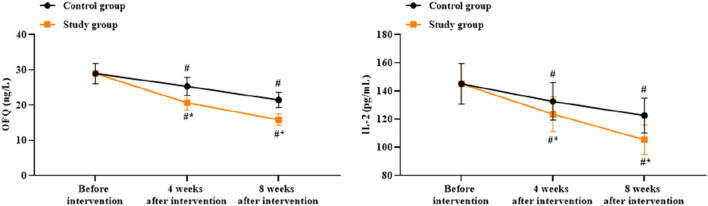
Serum levels of orphanin FQ (OFQ) and interleukin-2 (IL-2) in both groups. ^#^ *p* < 0.05 vs. before intervention; ^*^ *p* < 0.05 vs. control group.

There were no significant differences in the serum levels of OFQ and IL-2 between the two groups before intervention (*p* > 0.05). Compared to before intervention, the serum levels of OFQ and IL-2 of both groups were lower at 4 and 8 weeks after intervention (*p* < 0.05). Compared to the control group, the study group had lower serum levels of OFQ and IL-2 at 4 and 8 weeks after intervention (*p* < 0.05, Figure 8).

### Incidence of adverse reactions and nursing satisfaction between the two groups

There was no significant difference in the incidence of adverse reactions between the two groups (*p* > 0.05, Table 2). Compared to the control group, the study group reported better nursing satisfaction rate (*p* < 0.05, Table 2).

**TABLE 2:** Comparison of incidence of adverse reactions and nursing satisfaction between the two groups.

Item	Control group (n = 60)	Study group (n = 60)	χ^2^	P
Adverse reactions, n (%)				
Dizziness	1 (1.66)	2 (3.33)	0.15	0.69
Sleepiness	1 (1.66)	1 (1.66)		
Thirst	1 (1.66)	1 (1.66)		
Total incidence rate	3 (4.98)	4 (6.65)		
Nursing satisfaction, n (%)				
Very satisfied	24 (40.00)	30 (50.00)	5.92	0.01
Satisfied	26 (43.33)	28 (46.67)		
Dissatisfied	10 (16.67)	2 (3.33)		
Total satisfaction rate	50 (83.33)	58 (96.67)		

## Discussion

Long-term regular hemodialysis treatment often places a heavy psychological burden on patients with nephropathy, which frequently leads to negative emotions like anxiety, depression, and alexithymia that are unfavorable to the patients (Ebadi et al., 2021).

The main constituents of Shugan Jieyu capsule are *H. perforatum* and *A. senticosus*; of these, the latter has the effects of tonifying the liver and kidney, strengthening muscles and bones, and accelerating blood circulation, while the former can clear heat, drain dampness, soothe the liver, and resolve depression (Fan et al., 2021). Therefore, Shugan Jieyu capsule is clinically used to treat depression and neurasthenia (Kim et al., 2023). Modern pharmacological studies have shown that *H. perforatum* can enhance the activity of the drug metabolism enzyme CYP3A4 in the body to enhance the efflux transport function of P-glycoprotein and inhibit the reuptake ability of serotonin, thereby increasing the serotonin level (Tan et al., 2022). At present, there are many reports on the efficacy of Shugan Jieyu capsule in improving negative emotions (Liu et al., 2024; Wu et al., 2022).

Group psychological counseling is an interventional approach that has high appeal, high communication efficiency, and a broad range of communication components (Xu et al., 2022). It provides a good platform for hemodialysis patients so that they can communicate safely with and help each other to overcome difficulties (Sazesh et al., 2021). Patients enrolled in group therapy are provided several skills and means to solve problems through various forms of communication like symposia, education, interactive listening, case exchange, and presentation. Through barrier-free communication modes like role playing, listening to others, and being listened to, the patients not only gain understanding about others but also provide support and help to others, learn to better understand others, learn to express their emotions, and experience abilities relatively accurately.

Our findings demonstrate that the combined intervention led to significantly greater reductions in the TAS-20, HAMA, and HAMD scores in the study group compared to the control group. This aligns with and extends an earlier work that demonstrated the benefits of combined psychological and pharmacological approaches in this population. Notably, our study is a pilot effort to apply this specific integrative regimen—a standardized TCM formulation combined with structured group therapy—to target the core symptom of alexithymia. Our results suggest that Shugan Jieyu capsule treatment combined with group psychological counseling could relieve alexithymia, anxiety, and depression in patients undergoing hemodialysis. The potential action mechanism may involve the pharmacological activity of the Shugan Jieyu capsule on monoamine neurotransmitters that is synergistically combined with psychological skills training for emotional identification and communication provided by group counseling.

Long-term hemodialysis can easily lead to various complications that seriously influence the quality of life of patients; among these, sleep disorders are important factors affecting the quality of life of the patients. Our study results suggest that compared to before intervention, the PSQI scores were lower at 4 and 8 weeks after intervention and that the study group had lower PSQI scores than the control group at 4 and 8 weeks after intervention. These results imply that the combination of Shugan Jieyu capsule treatment and group psychological counseling could improve the sleep quality of hemodialysis patients. Similarly, it has been documented that the Shugan Jieyu capsule could improve sleep in patients convalescing from the effects of COVID-19 (Xuedong et al., 2022). Meanwhile, psychological intervention has been reported to be effective in improving the sleep quality of hemodialysis patients (Yangöz Ş and Özer, 2022). Our study provides additional evidence for the combined use of these two interventions in improving sleep quality in hemodialysis patients, which is a more comprehensive approach compared to single-intervention studies.

The combined intervention offered in our study led to significant reductions in the serum OFQ and IL-2 levels compared to the control group. This finding provides preliminary biochemical support for the efficacy of the proposed intervention. The decrease in OFQ aligns with the opioid peptide hypothesis, suggesting that the intervention may help normalize an overactive stress/emotional blunting pathway. Concurrently, the reduction in IL-2 level hints at a modulatory effect on the immunoinflammatory axis, potentially alleviating the inflammatory burden that exacerbates psychological distress and somatic preoccupation. Although correlational, the changes in these biomarkers lend mechanistic plausibility to the observed improvements in alexithymia and related psychological symptoms, moving beyond purely subjective scale-based assessments.

In addition, our study demonstrated that compared to before intervention, the PSP, ESCA, and WHOQOL-BREF scores were elevated at 4 and 8 weeks after intervention. Compared to the control group, the study group showed higher PSP, ESCA, and WHOQOL-BREF scores at 4 and 8 weeks after intervention. At the same time, compared to the control group, the study group reported better nursing satisfaction after intervention. These results indicate that the combination of Shugan Jieyu capsule treatment and group psychological counseling could improve the personal and social performances, self-care ability, quality of life, and nursing satisfaction in hemodialysis patients. Most of the previous studies have focused on either psychological interventions or TCM treatments alone (Taylor et al., 2023; Zhang et al., 2021), whereas the present study highlights the synergistic effects of combining these two approaches to achieve more comprehensive and effective improvements in multiple aspects of patient wellbeing.

*H. perforatum* has been known to potentially affect the metabolism of other drugs by inducing CYP3A4 and P-glycoprotein production (Nicolussi et al., 2020). In the present study, we ensured that none of the patients used any narrow therapeutic window drugs metabolized by CYP3A4 (such as warfarin or cyclosporine) concurrently and that the adverse reactions were closely monitored during the study period. The results showed no significant difference in the incidence of adverse reactions between the two groups, suggesting that the combined use under the current protocol is safe and controllable.

### Strengths, limitations, and future directions

Our study has several strengths, including the randomized controlled design, use of blinded outcome assessments, comprehensive set of validated psychological and biological outcome measures, and investigation of a novel multimodal intervention in a clinically challenging population.

However, several limitations should be acknowledged when interpreting our findings. First, although the procedures are assessor-blinded, we could not ensure double-blinding owing to the nature of the interventions, which may have introduced performance bias. Second, the sample population was recruited from a single center with the follow-up duration being limited to 8 weeks, which may affect the generalizability of our results and preclude conclusions about long-term sustainability of the effects. Third, the mechanisms underlying the observed reductions in OFQ and IL-2 levels remain speculative; future studies incorporating more comprehensive neurobiological and inflammatory marker panels are warranted for elucidating the precise pathways. Fourth, the group psychological counseling was a complex intervention; although we described the necessary protocols, the relative contributions of the specific components (e.g., psychoeducation, skills training, and peer support) to the overall effect cannot be disentangled in this design. Fifth, the choice of control intervention warrants careful consideration. The control group received estazolam, a benzodiazepine hypnotic with known anxiolytic properties. Although this provided an active comparator for sleep outcomes and reflected real-world clinical practices for symptomatic management, it introduced a potential pharmacological confounder when interpreting psychological outcomes, such as anxiety and depression to a lesser extent. The improvements seen in the control group for these measures are partially attributable to estazolam, which could lead to underestimation of the true effect size of our combined experimental intervention relative to a non-pharmacological or placebo control. Future efficacy trials should thus consider comparing the combined intervention against a first-line psychological therapy (e.g., CBT) or using a placebo capsule in the control group to isolate the specific effects of the TCM and counseling components clearly.

Despite these limitations, our findings provide promising preliminary evidence in support of the combination therapy. Future research should aim for larger multicenter trials with longer follow-up periods. Investigating the cost-effectiveness of the combined approach and exploring patient characteristics that better predict responses would also be valuable for clinical translation.

## Conclusion

The randomized controlled trial presented herein provides evidence that an 8-week intervention combining Shugan Jieyu capsule treatment with structured group psychological counseling is more effective than conventional therapy (estazolam and routine nursing) in alleviating alexithymia, anxiety, and depression in hemodialysis patients. The combined approach also led to significant improvements in the sleep quality, psychosocial functioning, self-care abilities, quality of life, and patient satisfaction and was additionally associated with favorable modulation of the serum OFQ and IL-2 levels. The interventions were well-tolerated, with a safety profile comparable to the control group. Although further confirmatory studies are needed, these preliminary results suggest that the integrative strategy holds promise as a valuable adjunctive treatment for addressing the complex psychological burden in this vulnerable patient population.

## Data Availability

The original contributions presented in the study are included in the article/Supplementary Material, and any further inquiries may be directed to the corresponding author.
